# FHIT overexpression in HepG2 hepatoma cells affects growth and cyclin D1 expression *in vitro*

**DOI:** 10.3892/etm.2013.1436

**Published:** 2013-12-04

**Authors:** JIAYUN GE, SIMIN SHEN, XIAOWEN ZHANG, KUN WANG, BO LIU, DEYUN SUN, LIN WANG

**Affiliations:** Department of Hepatobiliary Surgery, The Second Affiliated Hospital of Kunming Medical University, Kunming, Yunnan 650101, P.R. China

**Keywords:** hepatoma, fragile histidine triad, methylation, cyclin D1, signaling pathway

## Abstract

The aim of this study was to investigate the methylation status of fragile histidine triad (FHIT) and the effects of FHIT on cell growth and cyclin D1 expression in hepatoma cells. The total proteins from the human hepatoma cell lines HepG2, Hep3B and Huh7 were collected and the expression levels of FHIT were analyzed. The methylation status in the promoter region of FHIT in the hepatoma cells was measured using methylation-specific polymerase chain reaction (PCR). The HepG2, Hep3B and Huh7 cells were subsequently treated with 5-aza-2′-deoxycytidine (5-azadc) and the restoration of FHIT expression was then examined. A p-hemagglutinin (HA)-FHIT plasmid was constructed and used to transfect the HepG2 cells, and the inhibitory effects of the transfection on cell growth were then assessed. In addition, HepG2 cells were cotransfected with the pHA-FHIT plasmid and a cyclin D1 luciferase reporter plasmid, and the effects of FHIT on the activity of cyclin D1 transcription factor were analyzed using a luciferase assay. FHIT was observed to be expressed at a low level in Hep3B and HepG2 cells; however, it was expressed at a relatively high level in Huh7 cells. The promoter region of FHIT in the Hep3B and HepG2 cells was partially methylated, and 5-azadc treatment induced an increased expression of FHIT. The increased expression of FHIT inhibited the growth of HepG2 cells. Cotransfection with the pHA-FHIT plasmid significantly inhibited the transcriptional activity of the cyclin D1 promoter and decreased the expression of cyclin D1 in HepG2 cells. In conclusion, FHIT was partially methylated in the HepG2 and Hep3B hepatoma cells. The overexpression of FHIT inhibited cell growth and decreased the expression of cyclin D1 in HepG2 cells.

## Introduction

Hepatocellular carcinoma (HCC) is one of the most common tumors worldwide, and its incidence is unlikely to be reduced in the near future, despite the developments in surgical treatment. In China and Southeast Asia, the incidence of HCC is high, with HCC occurring most frequently following the development of chronic liver disease resulting from infection with hepatitis virus (1). Therefore, it is important to elucidate the molecular mechanism of HCC carcinogenesis and to develop specific measures for the prevention of HCC.

Fragile histidine triad (FHIT) protein belongs to the family of the evolutionary conserved histidine triad (HIT) proteins, which consists of at least three subfamilies: FHIT, histidine triad nucleotide binding protein 1 (HINT1) and galactose-1-phosphate uridylyltransferase (GALT) (2). Our previous study showed that HINT1 functions as a tumor-suppressor gene in human hepatoma cell lines (3). FHIT is located at human chromosome 3p14.2, and encodes a transcript of 1.1 kb (4). It encompasses the FRA3B fragile site and a genomic locus that is frequently involved in cytogenetic abnormalities, genomic rearrangement and allelic loss in tumors (5). The aberrant methylation of normally unmethylated CpG islands, located in the 5′ promoter region of genes, is associated with the transcriptional inactivation of a number of genes in human cancer and may serve as an alternative to mutational inactivation. The hyper-methylation of the 5′ promoter region of the FHIT tumor-suppressor gene has been observed in human cell lines of leukemia, as well as in breast, pancreatic and esophageal cancer (6–9). However, the methylation status of FHIT in human hepatoma cells requires further investigation.

The Wnt/β-catenin/transcription factor 4 (TCF4) signaling pathway is important in the survival, proliferation and differentiation of cells (10). Our previous study showed that in HepG2 human hepatoma cells, the HIT family member HINT1 binds to the Pontin/Reptin/β-catenin/TCF4 complex, and thus deactivates TCF4 (3). Mutations in the adenomatous polyposis coli (APC) gene have not been observed in experimental or human hepatoma. The increased expression of β-catenin has been correlated with a poor prognosis in patients with hepatoma. Following the activation of the Wnt/β-catenin/TCF4 pathway, cyclin D1 is induced to form an active enzyme complex with cyclin-dependent kinase 4/6 (CDK4/6), which phosphorylates and activates CDK and leads to the G1/S transition (11). As a result, cell proliferation and tumor genesis are induced. The activation of the Wnt/β-catenin/TCF4 pathway and the overexpression of cyclin D1 in human HCC cells have been studied previously (12,13).

The purpose of the present study was to examine the expression and function of FHIT in human hepatoma cells. These cells were the subject of the study due to the unusual role of β-catenin in hepatoma cells and the global prevalence of hepatoma.

## Materials and methods

### Cell lines and cell culture

The human hepatoma cell lines HepG2, Hep3B and Huh7 were obtained from the American Type Culture Collection (Manassas, VA, USA). The cell lines were maintained in a DF10 medium containing Dulbecco’s modified Eagle’s medium (DMEM; Invitrogen Life Technologies, Carlsbad, CA, USA) supplemented with 10% fetal bovine serum (FBS; Invitrogen Life Technologies), and were incubated in a 100% humidified incubator at 37°C with 5% CO_2_.

### Protein extraction and western blot analysis

The cells were cultured and treated as described above. The cells were collected and resuspended in 10 ml of lysis buffer (25 mM Tris-HCl, pH 7.5, 20 mM NaCl, 1 mM EDTA, 20% (v:v) glycerol, 1% (v:v)Triton X-100, 2X protease inhibitor mixture for proteins, respectively. After sonication (4×20 sec, 1 min between cycles), the lysates were centrifuged at 12,000 × g at 40°C for 20 min. The proteins were separated using SDS-PAGE with 7.5–12.5% polyacrylamide gels at 4°C for 2 h and subsequently blotted with the indicated antibodies at 4°C overnight. The primary antibodies included anti-FHIT, anti-hemagglutinin (HA)-Tag, anti-cyclin D1 and anti-β-actin (all from Sigma, St. Louis, MO, USA). Antimouse and antirabbit IgA (Amersham Biosciences, New York, NY, USA) antibodies were used as the secondary antibodies. Each membrane was developed with an enhanced chemiluminescence system (Amersham Biosciences). The intensities of specific protein bands were quantified with NIH Image software version 1.62 (NIH, Bethesda, MD, USA), corrected for the intensity of the respective β-actin band.

### Methylation-specific polymerase chain reaction (PCR)

Total cell DNA was isolated from the cultured cells using a QIAamp DNA Mini kit (Qiagen N.V., Venlo, The Netherlands). The genomic DNA was modified as described previously (14) using a DNA bisulfite modification kit (Chemicon, Temecula, CA, USA). The methylated CpG dinucleotides within a CpG island in the promoter region of FHIT were detected using the following specific PCR primer sets: methylated FHIT forward, 5-TTGGGGCGCGGGTTTGGGTTTTTACGC-3 and reverse, 5-CGTAAACGACGCCGACCCCACTA-3; and unmethylated FHIT forward, 5-TTGGGGTGTGGGTTT GGGTTTTTATG-3 and reverse, 5-CATAAACAACACCAACCCCACTA-3 (15). Each PCR mixture system contained 10X PCR buffer (Qiagen N.V.), deoxynucleotide triphosphates (1.25 mmol/l), primers (0.6 mmol/l), 1 unit HotStarTaq^®^ (Qiagen N.V.), and bisulfite-modified DNA (100 ng). PCR was performed in a programmable thermal controller (MJ Research, Inc., Quebec, Canada) under the following conditions: Preheating at 94°C for 3 min, 94°C for 30 sec, 62°C for 30 sec and 72°C for 30 sec for 38 cycles and a final extension at 72°C for 7 min. The PCR products were analyzed on a 2% agarose gel.

### 5-Aza-2′-deoxycytidine (5-azadc) treatment and FHIT expression

The cells were treated with a solution or with 5-azadc (1 μmol/l; Sigma) for 96 h. Following the extraction of total proteins from the cells, the expression levels of FHIT protein were examined using western blotting.

### Plasmid construction and gene transduction

Full-length human FHIT cDNA was amplified from HCT116 cells with a one-step reverse transcription (RT)-PCR procedure, using the primers 5-CCAATGGATCCATGTCGTTCAGATTTGG-3 (forward) and 5-CCAATCTCGAGTCACTGAAAGTAG ACC-3 (reverse) (16). The PCR products were then cloned into the *Bam*HI/*Xho*I-treated plasmid pHANE, which contained the NH_2_-terminal HA epitope tag (YPYDVPDYA) (14). The sequence of the pHA-FHIT construct was confirmed using DNA sequencing.

### Cell proliferation assays

#### Colony formation

HepG2 cells were transfected with the pHA-FHIT plasmid and the empty control plasmid pcDNA3. The cells were subsequently replated into 100-mm dishes at 1×10^5^ cells per dish, and were cultured in the presence of 0.8 mg/ml G418 for three weeks. The colonies were then fixed with formalin, stained with Giemsa and counted.

#### Growth curves

HepG2 cells were transfected with the PcDNA3 (Invitrogen Life Technologies) and HA-FHIT (constructed by Professor Lin Wang) plasmids and counted every day for eight days. The number of viable cells per well was determined each day. Each assay was performed in triplicate.

#### Luciferase reporter assays

The HepG2 cells were plated at 1×10^5^ cells per 35-mm-diameter plate 18 h prior to transfection. The cells were subsequently transfected with the p-1745-CD1-LUC luciferase reporter plasmid (400 ng/well) (and 200 ng/well p-cytomegalovirus (CMV)-β-galactosidase reporter plasmid, with or without cotransfection with HA-FHIT plasmid (400, 300, 200 or 100 ng) DNA. The p-1745-CD1-LUC, CMV-β-galactosidase and wild-type β-catenin plasmids were provided by Kathryn Calame (Columbia University, New York, NY, USA). At 36 h post-transfection, cell extracts were prepared, and each sample was assayed in triplicate, using a Luciferase assay system (Promega Corporation, Madison, WI, USA). Luciferase activities were normalized to β-galactosidase activities to correct for the differences in transfection efficiency. Significant differences (P<0.05) with respect to the corresponding control assay are indicated by an asterisk (*).

#### Statistical analysis

All data are presented as the mean ± standard deviation (SD). P<0.05 was considered to indicate a statistically significant difference. The statistical analysis was performed using SPSS version 11.0 statistical software (SPSS, Inc., Chicago, IL, USA).

## Results

### Expression of FHIT in human hepatoma cells

**Figure 1:**
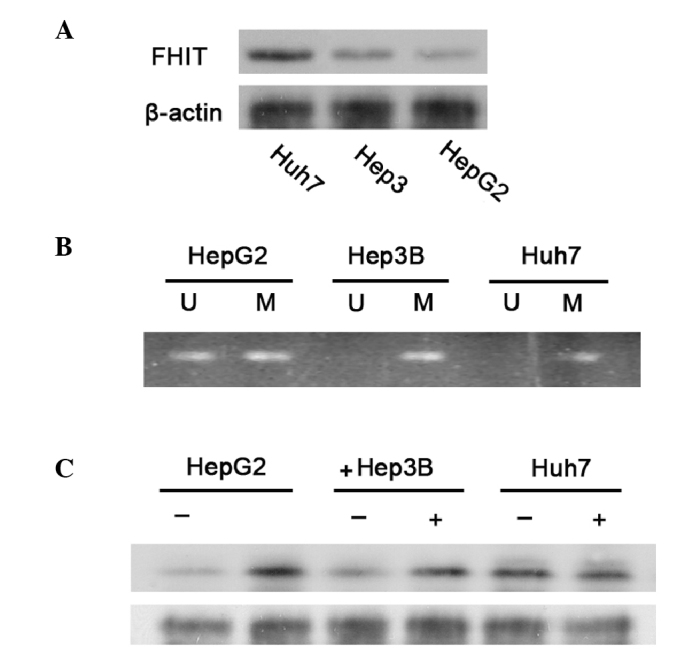
Fragile histidine triad (FHIT) protein expression and the methylation of the FHIT promoter in human hepatoma cell lines. (A) FHIT protein expression was examined using western blotting. (B) Methylation-specific polymerase chain reaction (PCR) was used to examine the methylation status of the FHIT promoter. Total cellular DNA was extracted, modified with bisulfite, and subjected to PCR with sets of specific PCR primers (U, unmethylated; M, methylated). The PCR products were analyzed on 1% agarose gels. (C) Cells were treated with 5-aza-2′-deoxycytidine (5-azadc; 1 μM) for 96 h. FHIT protein expression was examined using western blot analysis. (−) no treatment, (+) 5-azadc.

Western blotting indicated that FHIT protein was expressed at a low level in HepG2 and Hep3B cells; however, it was expressed at a relatively high level in Huh7 cells (Fig. 1A). Similar results were obtained in a repeated study.

### Methylation status of FHIT in hepatoma cells

The expression levels of certain specific tumor-suppressor genes may be inhibited in cancer cells via the methylation of cytidine residues in the corresponding promoter regions of these genes, or via other modifications that alter the structure of chromatin. Therefore, the methylation status of a CpG-rich region in the promoter region of FHIT was examined using bisulfite-treated DNA samples obtained from the three cell lines, with sets of methylation-specific primers. Partial methylation of the DNA was observed in the HepG2 and Hep3B cell DNA samples. No methylation of the DNA was found in Huh7 cells (Fig. 1B).

The results suggest that the relatively low levels of FHIT in HepG2 and Hep3B cells may have been at least partially due to the methylation of the promoter. Therefore, the three cell lines were treated with 1 μM 5-azadc, the DNA demethylation agent, for 96 h and then the protein expression levels of FHIT were examined using western blotting. In the HepG2 and Hep3B cells, treatment with 5-azadc led to a ~2-fold increase in the level of FHIT expression (Fig. 1C). By contrast, similar treatment with 5-azadc did not increase the FHIT expression levels in the Huh7 cells (Fig. 1C). Therefore, the results in Fig. 1 demonstrated that the relatively low-level expression of FHIT in HepG2 and Hep3B cells was at least in part due to the partial methylation of the promoter region of this gene.

### Increased expression of FHIT inhibits the growth of HepG2 cells

**Figure 2:**
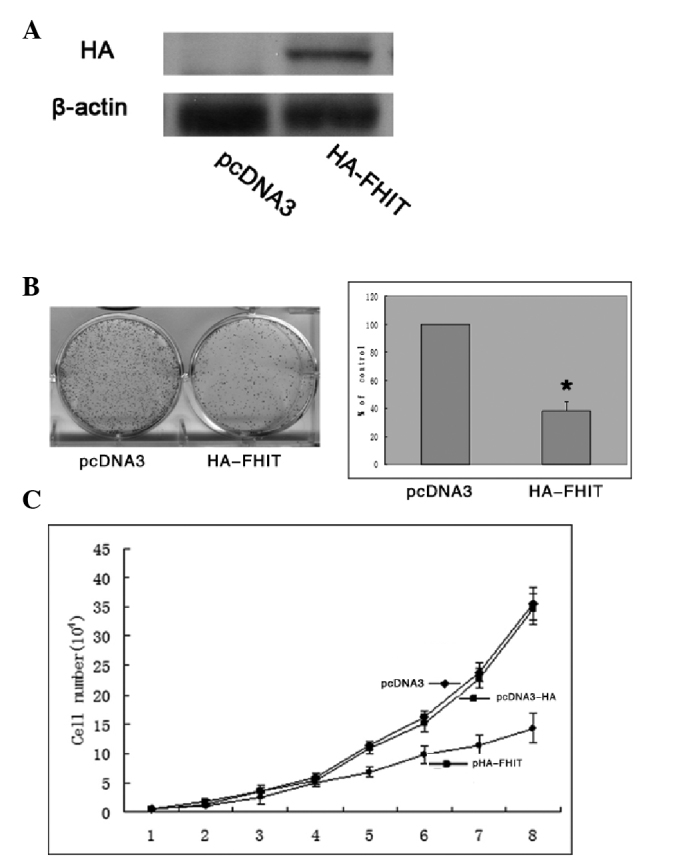
Fragile histidine triad (FHIT) overexpression inhibited cell growth in HepG2 cells. HepG2 cells were transfected with plasmid pcDNA3 encoding hemagglutinin (HA)-FHIT or empty pcDNA3. (A) Western blotting was used to examine the HA-FHIT expression. The transfected cells were grown in the presence of the selection agent G418 for three weeks. (B) The colonies were then fixed, stained with Giemsa solution and counted. The photographs show representative plates and the bar graph exhibits the percent inhibition of colony formation. The results are the mean ± standard deviation (SD) of triplicate assays. ^*^Significantly different from control (P<0.01). (C) Growth curves: Cells were transfected with pcDNA3-HA-FHIT, empty pcDNA3 or pcDNA3-HA and the number of cells in replicate wells was counted each day for the following eight days. The data are presented as the mean ± SD of triplicate assays.

A plasmid pHA-FHIT was constructed, which contained full-length FHIT open reading frame and an NH_2_-terminal HA epitope tag (YPYDVPDYA) (14). The constructed plasmid, pHA-FHIT, was confirmed by gene sequencing (data not shown). Subsequently, the plasmid encoding HA-FHIT or an empty control plasmid was used to transfect the HepG2 cell line. Transfection with HA-FHIT plasmid resulted in overexpression levels of the associated HA proteins (Fig. 2A). The overexpression of HA-FHIT inhibited colony formation by ~60% (P<0.05) compared with that of the control plasmid-transfected cells (Fig. 2B). In addition, cell proliferation assays were performed over a period of eight days, and cell proliferation was ultimately inhibited by ~50% in the HA-FHIT-transfected cells compared with the proliferation of the control plasmid-transfected cells (Fig. 2C).

### FHIT inhibits cyclin D1 expression in HepG2 cells

**Figure 3:**
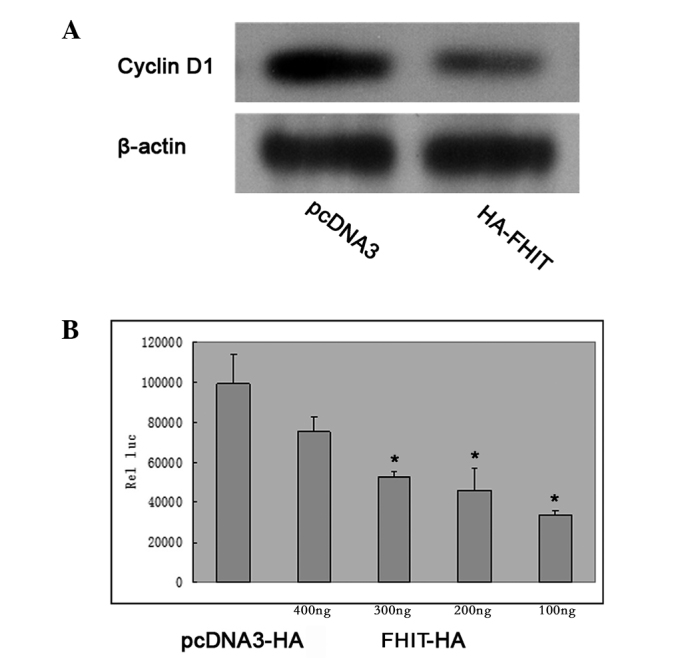
Fragile histidine triad (FHIT) inhibits cyclin D1 expression in HepG2 cells. (A) HepG2 cells were transfected with the indicated plasmid, prior to the cyclin D1 protein expression levels being examined using western blotting. (B) The full-length cyclin D1 promoter-luciferase reporter (p-1745-CD1-LUC) and cytomegalovirus (CMV)-driven β-galactosidase reporter plasmids were cotransfected into the HepG2 cells, with or without the indicated additional plasmids. Luciferase activity was assayed 36 h subsequent to transfection and normalized for β-galactosidase activity. HA, hemagglutinin. ^*^Significantly different from pcDNA3-HA.

Cyclin D1 is a protein involved in the cell cycle that is affected in the G1 phase of cell cycle. Western blotting of HepG2 cells transfected with HA-FHIT demonstrated that the overexpression of HA-FHIT inhibited the expression of cyclin D1 in the cells (Fig. 3A). In HepG2 cells which were transfected with a full-length cyclin D1 promoter-luciferase reporter (p-1745-CD1-LUC), cotransfection with increasing quantities of FHIT plasmid DNA caused a concentration-dependent inhibition of the transcriptional activity of the cyclin D1 promoter (Fig. 3B).

### FHIT inhibits the activity of cyclin D1 transcription factor through the β-catenin/TCF4 signaling pathway

**Figure 4:**
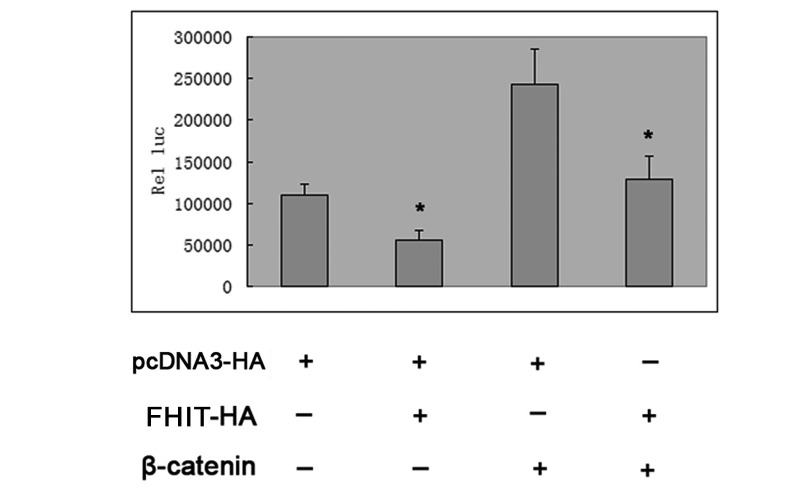
Fragile histidine triad (FHIT) inhibits the cyclin D1 transcription factor activity through the β-catenin/transcription factor 4 (TCF4) signaling pathway. The full-length cyclin D1 promoter-luciferase reporter (-1745-CD1-luc) and cytomegalovirus (CMV)-driven β-galactosidase reporter plasmids were cotransfected into HepG2 cells, with cotransfected FHIT-hemagglutinin (HA), pcDNA3-HA and/or wild-type β-catenin plasmid DNAs, as indicated. Luciferase activity was assayed 36 h subsequent to transfection and normalized for β-galactosidase activity. ^*^P<0.05 compared with FHIT-HA.

Transfection with plasmid DNA encoding wild-type β-catenin markedly activated the p-1745-CD1-LUC reporter in HepG2 cells; however, this activation was largely inhibited by additional cotransfection with FHIT plasmid DNA (Fig. 4). In combination, the results indicated that FHIT was capable of inhibiting the expression of cyclin D1 in HepG2 cells.

## Discussion

In view of the prevalence of HCC in Asia and Africa and the rising incidence and rates of mortality of HCC in the United States (1), the aim of the present study was to examine a number of molecular aspects of the function of FHIT in human hepatoma cells. A low-level expression of FHIT was observed in HepG2 and Hep3B cell lines compared with that of the Huh7 cell line (Fig. 1A). One key mechanism for the loss of function of tumor-suppressor genes in cancer involves gene silencing, mediated by hyper-methylation or aberrant promoter DNA. Previous studies demonstrated that the FHIT 5′ promoter region was partially methylated in certain human cancer cell lines (7,17–19). In the present study, methylation-specific PCR was used to show that the FHIT promoter was partially hyper-methylated in HepG2 and Hep3B cells (Fig. 1B), and that treatment with 5-azadc increased the expression of FHIT in these cells. Thus, it was concluded that FHIT was expressed at a low level in HepG2 and Hep3B cells, which was at least partially due to the hemi-methylation of the FHIT promoter in these cell lines.

Previous studies have demonstrated that the overexpression of FHIT, following transfection or infection of the cells with exogenous FHIT, may significantly inhibit cell growth and induce apoptosis in leukemia, as well as breast and esophageal cancer cell lines (20–23). In order to directly show that FHIT acts as a tumor-suppressor gene in hepatoma cells, the wild-type FHIT was generated and incorporated into an N′-terminal HA-tagged FHIT plasmid. Following transient transduction, FHIT-overexpressing cells were obtained. Colony formation and growth curve assays showed that the overexpression of exogenous FHIT significantly inhibited the growth of HepG2 cells (Fig. 2).

The HIT family is characterized by a common His-X-His-X-His-XX motif, in which X represents a hydrophobic amino acid (24). In our previous study, another HIT family member, HINT1, was demonstrated to bind to the Pontin/Reptin/β-catenin/TCF4 complex, inhibiting the activity of TCF4 and, thus, significantly inhibiting the expression of its direct substrate, cyclin D1. This activity of HINT1 was significant with regard to the cell cycle and cell proliferation (3). Therefore, it was of interest to investigate whether FHIT was also important in the β-catenin/TCF4 signaling pathway. When HepG2 cells were transfected with a full-length cyclin D1 promoter-luciferase reporter (p-1745-CD1-LUC), and cotransfected with increasing quantities of FHIT plasmid DNA, a concentration-dependent inhibition of the transcriptional activity of the cyclin D1 promoter was observed (Fig. 3B). Transfection with plasmid DNA encoding wild-type β-catenin markedly stimulated the activity of this reporter in HepG2 cells; however, this stimulatory activity was largely inhibited by additional cotransfection with FHIT plasmid DNA (Fig. 4). In order to directly examine the effects of FHIT on the expression of the endogenous cyclin D1 gene in HepG2 cells, these cells were transfected with pHA-FHIT, which encoded the FHIT cDNA sequence, (Fig. 2A). It was observed that overexpression of wild-type FHIT inhibited the expression of endogenous cyclin D1 protein in HepG2 cells.

The results of the present study indicate that FHIT is capable of directly inhibiting cell growth and suppressing cyclin D1 expression in HepG2 cells. These observations provide a rationale for targeting FHIT and the associated pathways as a novel approach for chemoprevention and cancer therapy. Additional studies are required to examine the clinical relevance of impairments in the expression and function of FHIT with respect to HCC and other types of human cancer.
